# Fast and SNP-aware short read alignment with SALT

**DOI:** 10.1186/s12859-021-04088-6

**Published:** 2021-08-25

**Authors:** Wei Quan, Bo Liu, Yadong Wang

**Affiliations:** School of Computer Science and Technology, Harbin Institute of Technology, 92 West Dazhi Street, Harbin, China

**Keywords:** NGS, Alignment, SNP-aware

## Abstract

**Background:**

DNA sequence alignment is a common first step in most applications of high-throughput sequencing technologies. The accuracy of sequence alignments directly affects the accuracy of downstream analyses, such as variant calling and quantitative analysis of transcriptome; therefore, rapidly and accurately mapping reads to a reference genome is a significant topic in bioinformatics. Conventional DNA read aligners map reads to a linear reference genome (such as the GRCh38 primary assembly). However, such a linear reference genome represents the genome of only one or a few individuals and thus lacks information on variations in the population. This limitation can introduce bias and impact the sensitivity and accuracy of mapping. Recently, a number of aligners have begun to map reads to populations of genomes, which can be represented by a reference genome and a large number of genetic variants. However, compared to linear reference aligners, an aligner that can store and index all genetic variants has a high cost in memory (RAM) space and leads to extremely long run time. Aligning reads to a graph-model-based index that includes all types of variants is ultimately an NP-hard problem in theory. By contrast, considering only single nucleotide polymorphism (SNP) information will reduce the complexity of the index and improve the speed of sequence alignment.

**Results:**

The SNP-aware alignment tool (SALT) is a fast, memory-efficient, and SNP-aware short read alignment tool. SALT uses 5.8 GB of RAM to index a human reference genome (GRCh38) and incorporates 12.8M UCSC common SNPs. Compared with a state-of-the-art aligner, SALT has a similar speed but higher accuracy.

**Conclusions:**

Herein, we present an SNP-aware alignment tool (SALT) that aligns reads to a reference genome that incorporates an SNP database. We benchmarked SALT using simulated and real datasets. The results demonstrate that SALT can efficiently map reads to the reference genome with significantly improved accuracy. Incorporating SNP information can improve the accuracy of read alignment and can reveal novel variants. The source code is freely available at https://github.com/weiquan/SALT.

## Background

Advancements in next-generation sequencing technologies open opportunities to various biological analyses, such as de novo assemblies of bacterial and eukaryotic genomes, and species classification based on metagenomics studies [1]. Short read alignment is a common first step of various downstream analyses, such as variant calling [2], RNA abundance quantification [3], and expression quantitative trait locus (eQTL) analysis [4].

It plays a critical role in medical and population genetics. Conventional aligners map sequencing reads to a linear reference genome, which represents one or a few individuals. However, such a linear reference genome lacks information on the variation in the population and consequently does not reflect the genetic diversity of individuals.

Augmenting the reference genome with known genetic variants can reduce the genetic distance between the donor and reference genomes and avoid allelic bias [5].

More than a decade ago, several short read alignment tools, such as BWA [6, 7], SOAP2 [8], and Bowtie [9, 10], were developed to map short reads to a linear reference genome efficiently. Through the adaptation of the Burrows–Wheeler transform (BWT) [11], these methods perform efficient alignment to a linear reference genome [12] in only a limited amount of memory [13, 14]. These aligners typically build an FM-index of a single reference genome and then use a variant of the backward search algorithm to find occurrences of sequencing reads in the reference genome. However, sequencing errors and genomic variants between the reference genome and sequencing reads may lead to incorrect alignments. Mapping sequencing reads to a single human reference genome leads to inherent biases towards the arbitrarily chosen reference.

Recently, several variant-aware aligners have been developed. BWBBLE [15] builds an FM-index for the expanded reference, which extends the alphabet from the 4-letter nucleotide code to the 16-letter IUPAC nucleotide code. Vg [16] uses the GCSA2 [17] graph indexing library to represent genetic variations as a bidirected sequence graph in its index.

HISAT2 modify the hierarchical indexing scheme from HISAT to create a hierarchical graph FM-index, which is combined with GCSA [18] to align DNA and RNA sequences.

Compared to conventional sequence alignment tools, genomic variant-aware aligners can reduce the differences between the reference and donor genomes, which leads to better alignment accuracy. However, the ability to store and index various types of variations requires precious RAM space, and aligning reads to a graph-based index is not as efficient as conventional aligners. Among all types of genomic variants, single nucleotide polymorphisms (SNPs) are approximately ten times as numerous as others.

Accordingly, indexing only the SNP information can not only improve the accuracy of alignment but also enable alignment with low memory requirements.

In this article, we present SALT, a BWT-based short read aligner that incorporates genetic SNPs to augment the reference genome. It can effectively map reads to a reference genome with low memory requirements. We have benchmarked SALT on both a simulated dataset representing known variations and a real sequencing dataset with UCSC Common SNPs set. The results show that SALT can achieve higher accuracy and sensitivity than aligners that do not incorporate variation information. Furthermore, SALT is very efficient to map short reads and has only a small memory footprint. We believe that SALT, as an SNP-aware read alignment algorithm, has enormous potential in variant calling and other downstream biological analyses.

## Results

### Implementation

We have implemented SALT to align short reads to a reference genome and SNP database. It performs alignment for both single-end and paired-end reads and allows multithreading. The default output format is SAM [19]. SALT is distributed under the GNU General Public License (GPL). The source code is available at https://github.com/weiquan/SALT.

The performance of SALT has been compared with that of the most widely used alignment tool, BWA-MEM (version 0.7.17-r1188). The aligners were tested on two simulated datasets and two high-throughput sequencing (HTS) datasets to assess their speed, sensitivity, and accuracy. All benchmarks were conducted on a desktop computer with 32 GB of RAM and a 3.30 GHz Intel i9-7900X processor with a total of 10 CPU cores running Linux Ubuntu 18.04.

### Evaluation on simulated datasets

We simulated 4 million 100 bp and 150 bp Illumina-like reads from the human genome GRCh38 using Mason2 [20] with a 0.1% SNP mutation rate, a 0.02% indel mutation rate and a 0.4% average sequencing error rate. We ran two versions of SALT, namely, SALT.snp and SALT.linear, which use an SNP-aware index and a linear reference index, respectively, for read alignment. Both SALT.snp and SALT.linear extended seeds with snpLV.

Table 1 shows the alignment results of all aligners for the 100 bp and 150 bp datasets. SALT.snp indexes human genome reference GRCh38 and 2.9M SNPs (simulated using Mason2 with the default settings). SALT.linear indexes only human genome reference GRCh38. We used the sensitivity, accuracy and running time to estimate the performance of read alignments on simulated datasets. A read is considered to present a perfect alignment (*PA*) if its best location is within a distance of 4 bp to the original coordinate. Given a dataset with *N* reads and *n* out of *N* are mapped, the sensitivity and accuracy are defined as follows:

$$\begin{aligned} Sen = & \#PA/n \times 100\%\\ Acc = & n / N \times 100\% \end{aligned}$$

**Table 1:** Statistics on simulated human datasets

Aligner	Arg	Sen (%)	Acc (%)	Uniq (%)	Unmapped	T1	T4
dataset 1: sim-70							
SALT.snp	-r 21	99.48	94.15	86.87	20,803	**8 m 56 s**	**2 m 12 s**
	-r 10	99.50	94.18	86.69	19,922	10 m 26 s	2 m 29 s
	-r 5	99.54	**94.24**	86.49	18,382	12 m 56 s	3 m 1 s
SALT.linear	-r 21	99.46	94.15	86.88	21,682	9 m 54 s	2 m 23 s
	-r 10	99.48	94.18	86.71	20,602	10 m 14 s	2 m 38 s
	-r 5	99.53	94.23	86.52	18,617	11 m 59 s	2 m 38 s
BWA-MEM		**100.00**	93.83	**92.29**	**60**	11 m 56 s	2 m 59 s
dataset 1: sim-100							
SALT.snp	-r 21	99.68	95.67	90.74	12,667	9 m 52 s	2 m 32 s
	-r 10	99.70	95.71	90.59	12,022	12 m 6 s	3 m4 s
	-r 5	99.70	**95.72**	90.51	11,829	15 m 4 s	3 m 51 s
SALT.linear	-r 21	99.68	95.67	90.78	12,817	**9 m 6 s**	**2 m 23 s**
	-r 10	99.70	95.71	90.64	12,067	10 m 51 s	2 m 48 s
	-r 5	99.70	**95.72**	90.56	11,856	13 m 3 s	3 m 32 s
BWA-MEM		**100.00**	95.34	**93.54**	**1**	14 m 11 s	3 m 36 s
dataset 2: sim-150							
SALT.snp	-r 21	99.86	96.72	92.21	5448	**12 m 52 s**	3 m 20 s
	-r 10	99.87	**96.73**	92.22	5235	16 m 4 s	4 m 9 s
	-r 5	99.87	**96.73**	92.15	5151	21 m 16 s	5 m 25 s
SALT.linear	-r 21	99.86	96.72	92.32	5482	13 m 30 s	**3 m 6 s**
	-r 10	99.87	96.72	92.34	5265	14 m 19 s	3 m 43 s
	-r 5	99.87	**96.73**	92.28	5179	18 m 4 s	4 m 40 s
BWA-MEM		**100.00**	96.35	**94.36**	**0**	20 m 20 s	5 m 35 s

SALT.linear and SALT.snp seed alignments with a linear reference and an SNP-augmented reference, respectively

SALT selects k-mers in reads every *X* nt with the setting “-r X”

T1 and T4 are escaped time with 1 thread and 4 threads, respectively

Best results are shown in bold

**Fig. 1:**
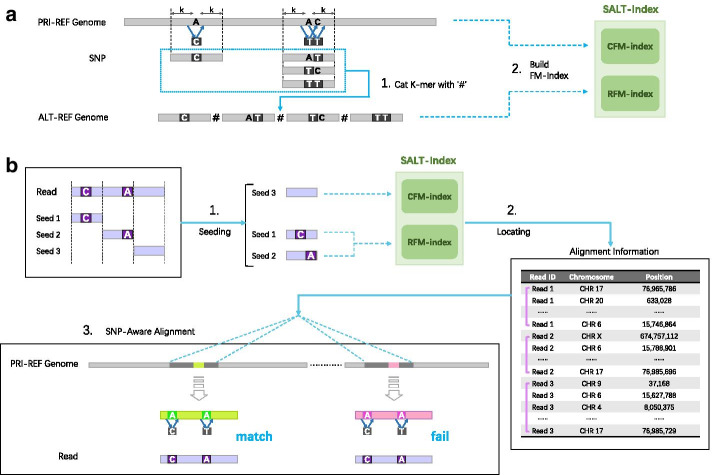
An example where the linear reference aligner fail to achieve satisfactory sequence alignment

Concerning accuracy, regardless of the parameters, both SALT.linear and SALT.snp are more accurate than BWA. An example where BWA-MEM fail to achieve satisfactory sequence alignment is shown in Fig. 1. SALT.snp differs from SALT.linear by no more than 0.01% with the same argument. In the case of the same index, whether the seed has an overlap can lead to a seeding effect of up to 0.09%.

Concerning speed, SALT.linear with the setting “-r 21” and the setting “-r 10” runs faster than BWA-MEM on all simulated datasets.

Concerning memory, the peak memory usage levels of SALT.snp and BWA are 5.09 GB and 5.24 GB, respectively. Sufficient memory for both is available on most desktop and laptop computers.

### Evaluation on HTS datasets

To assess the performance on real data, we benchmarked all aligners on two real datasets. Four million 100 bp reads sequenced with the Illumina HiSeq 2000 (SRA ID: ERR037900) and four million 148 bp reads sequenced with the Illumina HiSeq 2000 (SRA ID: SRR1766443) were mapped to the human reference genome (GRCh38).

We ran two versions of SALT, namely, SALT.snp and SALT.linear, which use an index incorporating 12.8M UCSC Common SNPs (build 151) and the original FM-index, respectively. UCSC Common SNPs is a subset of dbsnp that have a minor allele frequency (MAF) of at least 1% and are mapped to a single location in the reference genome assembly.

SALT was run with different overlap lengths in the seeding phase, leading to differences in speed and accuracy. BWA-MEM was run with the default settings.

Because the exact locations of the reads in the reference genome were not known, we assumed only that the reads should be mapped to the most similar locations in the human reference genome. Therefore, a read is considered a perfect alignment (*PA*) if the edit distance (including clipping) between the read and the reference was less than 10% of the read length. The results are shown in Table 2.

**Table 2:** Statistics on real human datasets

Aligner	Arg	Sen (%)	Acc (%)	Uniq (%)	Unmapped	T1	T4
dataset 1: real-100							
SALT.snp	-r 21	99.15	**99.76**	90.43	33,981	9 m 37 s	2 m 37 s
	-r 10	99.24	**99.76**	90.19	30,341	11 m 41 s	3 m 8 s
	-r 5	99.27	**99.76**	90.09	29,380	13 m 24 s	3 m 38 s
SALT.linear	-r 21	99.13	**99.76**	90.51		**9 m 30 s**	**2 m 26 s**
	-r 10	99.23	**99.76**	90.29	30,619	10 m 34 s	2 m 52 s
	-r 5	99.26	**99.76**	90.21	29,478	11 m 37 s	3 m 16 s
BWA-MEM		**99.99**	97.59	**91.60**	**374**	18 m 59 s	5 m 42 s
dataset 2: real-148							
SALT.snp	-r 21	97.39	99.95	89.35	104,347	18 m 13 s	**4 m 35 s**
	-r 10	97.44	99.94	89.30	102,474	19 m 45 s	6 m 23 s
	-r 5	97.47	99.94	89.21	101,457	26 m 34 s	7 m 25 s
SALT.linear	-r 21	97.38	**99.96**	89.49	104,839	**14 m 14 s**	3 m 47 s
	-r 10	97.43	99.95	89.47	102,905	19 m 38 s	5 m 12 s
	-r 5	97.46	99.95	89.39	101,795	25 m 20 s	6 m 40 s
BWA-MEM		**99.51**	97.28	**93.55**	**19,450**	23 m 18 s	6 m 29 s

SALT.linear and SALT.snp seed alignments with a linear reference and an SNP-augmented reference, respectively

SALT selects seeds in reads every *X* nt with the setting “-r X”

T1 and T4 are escaped time with 1 thread and 4 threads, respectively

Best results are shown in bold

In this evaluation, BWA-MEM is the most sensitive. Both SALT.linear and SALT.snp are more accurate than BWA-MEM. With the same argument, SALT.snp is slightly more sensitive and accurate than SALT.linear. As the argument *X* increases, the difference in sensitivity and accuracy between SALT.snp and SALT.linear decreases.

SALT.linear with the argument $X=21$ is the fastest on both 100 bp and 148 bp real dataset.

Concerning memory, BWA uses 5.24 GB. Both SALT.snp and SALT.linear use 5.81 GB. Hence, memory is not a practical concern with either BWA or SALT; both can be run on most desktop computers.

## Discussion and conclusion

In this article, we proposed a compact representation for an augmented reference genome that combines a human reference genome with genomic SNPs. We presented a novel index to support SNP-aware searches, designed an SNP-aware seeding algorithm, modified the Landau–Vishkin and Smith–Waterman algorithms to support SNP-aware pairwise alignment, and implemented a short read alignment tool (SALT). SALT is a BWT-based short-read aligner that incorporates SNPs. Evaluations on both simulated and real data suggest that SALT based on a human reference genome incorporating 12.8M UCSC common SNPs (build 151) offers improved accuracy. SALT does not output alignments with edit distances greater than 10% of the read length, which leads to lower sensitivity than BWA-MEM. However, such alignments would be more likely to result in wrong alignments. Although aligning reads to an SNP-augmented reference is slightly slower than alignment to a linear human genome in some cases, it is considerably more efficient when aligning reads to the major histocompatibility complex (MHC) region. Moreover, SALT is naturally scalable to the alignment of reads that span more complicated genomic variants through the extraction of sequences nearby those genomic variants, and its utility will increase once large databases of genomic variants are available. Finally, SALT can output information about the SNPs in datasets (e.g., the SNP ID number), which would simplify and improve the current post-alignment processing pipeline.

Overall, SALT is a fast, memory-efficient and SNP-aware short read alignment tool. This method shows enormous potential in variant calling and complex variation detection for a large population of genomes.

## Methods

### Overview of the SALT approach

A linear reference genome and a set of SNPs can be represented as a graph genome by iteratively adding edges corresponding to non-reference alleles and terminating at nodes corresponding to genomic loci on the initial edge [21]. A graph genome can be indexed by creating a hash table with k-mers along all possible paths of the graph. However, building such a hash-table-based index for the human genome usually requires a large amount of memory (i.e., >12 GB for a linear human reference genome). While the size of the hash table can be reduced by sampling the k-mers every *x* nt, this process will potentially decrease the number of seed matches, which will reduce the accuracy and sensitivity of the sequence alignment. Therefore, a compressed data structure is needed to index a graph genome.

Ferragina and Manzini first introduced the FM-index data structure in 2000. This structure extends the BWT representation of a string by adding suffix array (SA) and character occurrence (OCC) data structures. An FM-index is a compressed representation of a string that requires considerable memory and supports searching for text within a rather low search time. We apply a modified BWT-based index (SALT-index) to index k-mers along all possible paths of a graph.

We define the alleles in the linear reference genome as the primary alleles and alleles that are not in the linear reference genome as alternative alleles. K-mers without alleles or with primary alleles can be indexed by the FM-index constructed for a standard linear reference genome, which is called the CFM-index. in section 2.2, we show that k-mers with alternative alleles can also be indexed by a variation of the FM-index. Thus, we propose an algorithm for SNP-aware alignment based on this index.

SALT implements the SNP-aware alignment in four main steps, as follows:

1. concatenate all genomic sequences around SNPs with the symbol $\#$ to generate an alternative reference;
2. build the FM-index for the primary reference, which is called the CFM-index, and the FM-index for the alternative reference, which is called the RFM-index;
3. generate maximal exact match (MEM) seeds based on the CFM-index and RFM-index and choose candidate alignment locations;
4. perform SNP-aware pairwise alignment between the reference and reads and report the possible alignments.

**Fig. 2:**
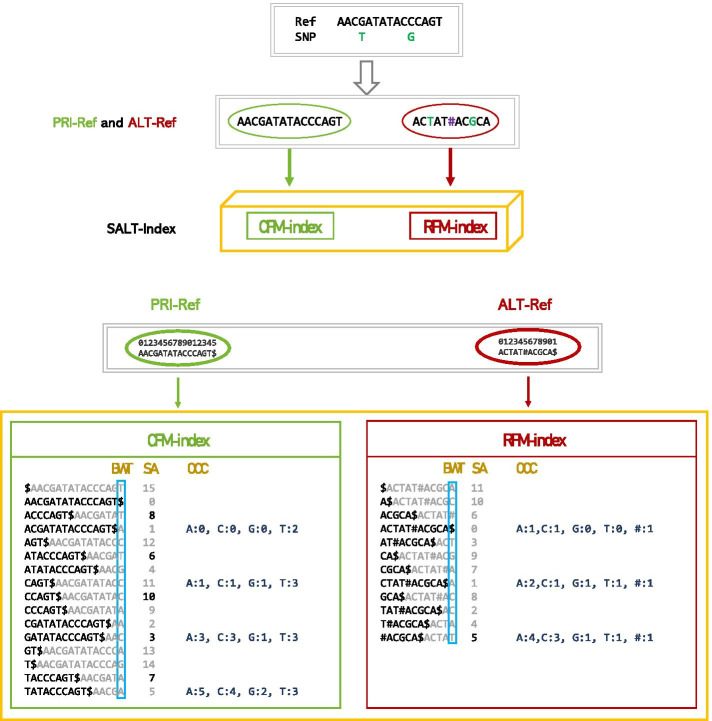
A flowchart of SALT-index construction and read alignment

A flowchart of index construction and read alignment is shown in Fig. 2.

### Construction of the SALT-index

SALT implements a BWT-based index (SALT-index) and an alignment algorithm to achieve fast and sensitive alignment of reads with respect to a reference genome and a large collection of SNPs. In contrast to other BWT-based aligners, our algorithm employs two different types of indices: (1) a four-alphabet FM-index of the primary reference genome (PRI-REF) that represents all k-mers without alternative alleles; and (2) a five-alphabet FM-index of the alternative reference genome (ALT-REF) that represents k-mers with alternative alleles.

**Fig. 3:**
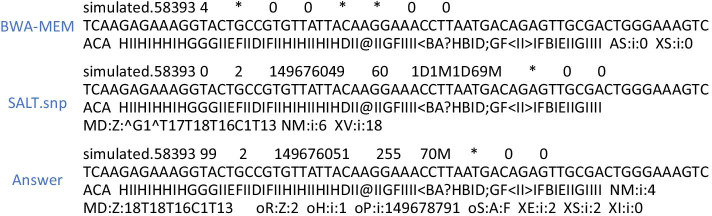
A flowchart of SALT-index construction

By building the FM-index for the primary reference genome sequence, all k-mers without alternative SNPs are indexed (CFM-index). To retrieve k-mers containing alternative SNPs, we enumerate all possible sequences in the range $[P-k, P + k]$ on the reference centered on SNP site *p* and concatenate all sequences with $\#$, called ALT-REF. BWT and OCC are built for ALT-REF (the size of the alphabet is 5). The starting positions of the suffixes need to be stored in the SA to transform the SA intervals into positions within the primary reference genome coordinates, and only sampled positions are usually stored to save memory space. However, the positions stored in a conventional SA are not defined in the primary reference genome coordinates. Here, we store all starting positions of suffixes in ALT-REF beginning with $\#$ in the SA in the primary reference genome coordinates. The index of ALT-REF, which combines BWT, OCC and the modified SA, is called the RFM-index. Adding known variations to the reference results in a structure that can be described as a mixed index (SALT-index), that consists of the CFM-index and the RFM-index. We demonstrate that, compared to an existing linear reference genome (GRCh38), the SALT-index can substantially improve the fraction of reads that are mapped uniquely and perfectly. A flowchart of SALT-index construction process is shown in Fig. 3.

To perform SNP-aware pairwise alignments, we use a four-bit Gray code to encode the alleles at each site on the enhanced genome reference as introduced in BWBBLE [15]. Each bit of the Gray code corresponds to a given nucleotide and is set to 1 if the given nucleotide is an allele at this site; otherwise, it is set to 0. For instance, the Gray code 0011 means that the alleles at the corresponding site are G and T.

### SNP-aware alignment via the SALT-index

SALT utilises a typical seed-extension strategy, and the algorithm for the alignment is shown in Algorithm 1.

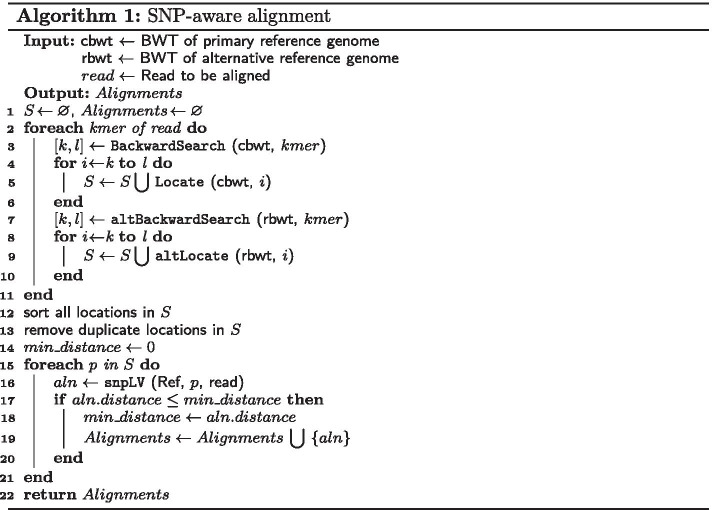



### SNP-aware seeding based on the SALT-index

The alignment process requires the identification of all SNP-aware seeds. If a SNP is not contained in a seed, then the candidate location of the seed can be found in the CFM-index. If a seed does contain an SNP, then the seed can be found in the RFM-index (which contains all possible alt-sequences containing SNPs). In the seeding phase, for any seed that does not contain any sequencing errors or contains a known SNP, we need to determine its location in the primary reference genome and use it as a candidate for SNP-aware pairwise alignment. We first search for the seed *s* in the CFM-index and obtain all occurrences of *s* in the primary reference. Then, we search for the seed *s* in the RFM-index (see Algorithm 2) and obtain all occurrences of *s* in the alternative reference,. The *altLocate* function for the RFM-index is presented in Algorithm 3.

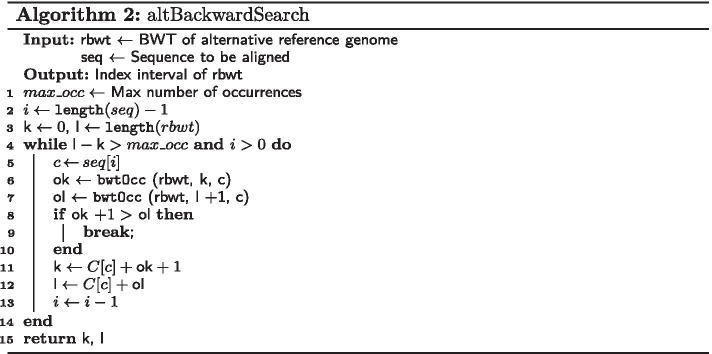

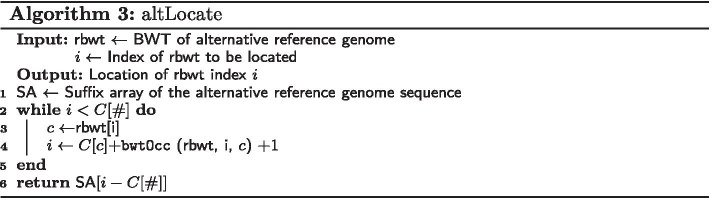





### Extension with SNP-aware Landau–Vishkin alignment

At this stage, we perform pairwise alignment between the read sequence and the reference genome sequence at the candidate locations to calculate pairwise alignment scores and choose the highest score location as the best alignment result. SALT supports two pairwise alignment algorithms, namely, snpLV and snpSW. In snpLV, the edit distance is used as the measure of sequence similarity (known SNPs do not increase the edit distance).

We adapt the Landau–Vishkin algorithm [22], which is an efficient string matching algorithm, to implement snpLV. We use four-bit Gray codes to encode the read sequence, which is denoted by $Q_g$. In a similar way, the Gray-encoded sequence in the augmented reference genome that begins with the candidate location is denoted by $T_g$.

The recurrence formula [23] for Landau–Vishkin alignment is as follows:

1 $$\begin{aligned} L_{d,e} = \max {\left\{ \begin{array}{ll} L_{d,e-1} +1&{} \quad + LCE(L_{d,e-1}+2, L_{d,e-1}+d+2)\\ L_{d-1,e-1} &{} \quad + LCE(L_{d-1,e-1}+1, L_{d-1,e-1}+d+1)\\ L_{d+1,e-1} +1&{} \quad + LCE(L_{d+1,e-1}+2, L_{d+1,e-1}+d+2) \end{array}\right. } \end{aligned}$$

We propose a SNP-aware longest common extension (LCE) algorithm to perform SNP-aware Landau–Vishkin alignment (Algorithm 4).

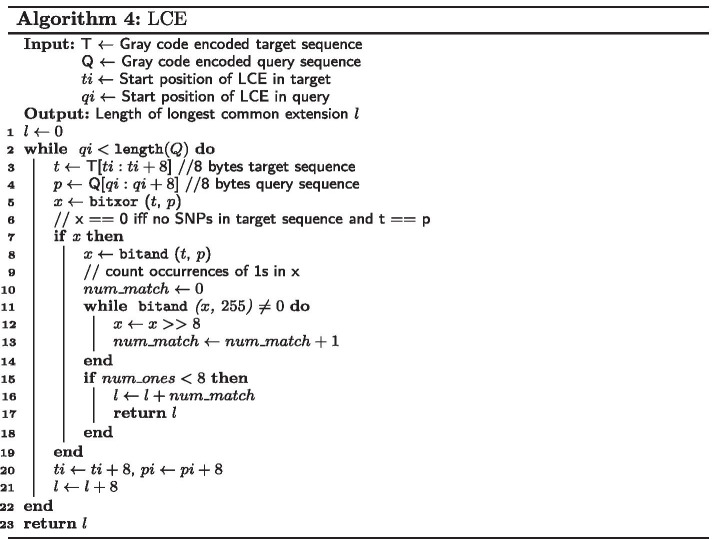



#### Extension with SNP-aware Smith–Waterman alignment

We adapt the Smith–Waterman algorithm [24] to implement the snpSW. The recurrence formulas for the Smith–Waterman algorithm [24] with the Gotoh improvements [25] for affine gap penalty functions are shown below.

2 $$\begin{aligned}&{\left\{ \begin{array}{ll} H_{i,j} &{}= \max \{H_{i-1,j-1}+S(i, j), E_{i,j}, F_{i,j}, 0\}\\ E_{i,j} &{}= \max \{H_{i,j-1}-o, E_{i,j-1}\} -e\\ F_{i,j} &{}= \max \{H_{i-1,j}-o, F_{i-1,j}\} -e\\ \end{array}\right. } \end{aligned}$$

3 $$\begin{aligned} S(i, j)&= {\left\{ \begin{array}{ll} a &{} \text {if T[ i] = Q[ j]}\\ b &{} \text {otherwise} \end{array}\right. } \end{aligned}$$

where *o* and *e* are the gap opening penalty and gap extension penalty, respectively; *S* is the substitution matrix, which describes the substitution penalty when one character in a sequence is changed to another character; *S*(*i*, *j*) is the substitution penalty for target sequence *T*[*i*] and query sequence *Q*[*j*]; and *a* and *b* are the match penalty and mismatch penalty, respectively. The formulas given above can be adapted for SNP-aware local alignment by modifying the substitution matrix. The modified substitution matrix is defined as follows:

4 $$\begin{aligned} S(i, j) = {\left\{ \begin{array}{ll} a &{} \text {if} \quad T_g[i] \& Q_g[j] = 1\\ b &{} \text {otherwise} \end{array}\right. } \end{aligned}$$

where $T_g[i]$ and $Q_g[j]$ are the four-bit Gray code encoded target sequence and query sequence, respectively. For a more efficient implementation, the substitution matrix in the SSW library [26] is modified to perform SNP-aware local alignment.

### Other practical concerns

#### Paired-end mapping

Given the *i*th hit for the first read and the *j*th hit for the second read, SALT computes their distance $d_{i,j}$.

If the two hits have the correct orientation and $d_{i,j}$ is in the interval $[min\_distance, max\_distance]$, then SALT reports a paired alignment; otherwise, it reports an unpaired alignment.

The values of $min\_distance$ and $max\_distance$ are the minimum and maximum distance between two ends of reads, which are generally set to $u-3\sigma$ and $u+3\sigma$, respectively. If a mate read is unmapped, SALT performs the Smith–Waterman alignment [26] for the mate in the interval $[min\_distance, max\_distance]$.

#### Refining alignments

SALT outputs the alignments of the reads mapped to the SNP-enhanced reference. SALT considers identical penalties for primary alleles and alternative alleles.

However, some downstream analyses are based on linear genomes, which requires realigning all hits to the primary reference. We present a refinement program (Polish) to realign all hits to the linear reference genome and compute the optimal alignment using the standard Smith–Waterman algorithm. The realignment results are stored in SAM format.

## Data Availability

Reference genome GRCh38 is available at ftp://ftp.ensembl.org/pub/release-84/fasta/homo_sapiens/dna. SNPs is available at http://hgdownload.cse.ucsc.edu/goldenPath/hg38/database/151Common.txt. Mason2 is available at https://github.com/seqan/seqan/tree/master/apps/mason2. ERR037900 and SRR1766443 are available at https://www.ncbi.nlm.nih.gov/sra.

## Abbreviations

NGS: Next-generation sequencing
SNP: Single nucleotide polymorphism
Arg: Argument
Sen: Sensitivity
Acc: Accuracy
Uniq: Unique
